# Supplementary material for: The Prevalence of Metabolic Syndrome and Its Components in Firefighters: A Systematic Review and Meta-Analysis
Source: Int J Environ Res Public Health. 2023 Sep 23;20(19):6814. doi: 10.3390/ijerph20196814 (PMC10572458; doi:10.3390/ijerph20196814)
Supplement: Supplementary file 1 [file ijerph-20-06814-s001.zip › Supplementary Table S1. Study and participant characteristics .pdf]

Metabolic syndrome prevalence in firefighters: A systematic Review and Meta-analysis

Supplementary Table S1: Summary of study and participant characteristics in included studies

Author	Design	Country	Male (%)	Diagnosis tool	Age (y)	Cases of Metsyn	Total sample size	MetSyn (%)	BMI (kg.m^2)	BP (mmHg)	TG (mg/dL)	FBG (mg/dL)	HDL (mg/dL)	WC (cm)	RoB Score (/100)
Baur *et al.*, (2012a)	Cross sectional	USA	100	JIS	39.6 ± 8.5	213	768	28	-	-	-	-	-	-	67
Baur *et al.*, (2012b)	Cross sectional	USA	100	JIS	37.6 ± 8.5	271	957	28	29.4 ± 4	123/80	195 ± 39	94 ± 21	45 ± 11	-	78
Bode *et al.*, (2021)	Cross sectional	USA	99	NCEP/ ATPIII	40.6 ± 1.8	445	4453	10	-	-	-	-	-	-	100
Carey *et al.*, (2011)	Longitudinal	USA	97	NCEP/ ATPIII	42 ± 7.5	35	75	47	29.9 ± 3	139/88	155 ± 86	119 ± 15	41 ± 17	104 ± 11	67
Choi *et al.*, (2017)	Cross sectional	USA	97.6	NCEP/ ATPIII	42.7 ± 8.9	41	288	14	-	-	-	-	-	-	100
Chung *et al.*, (2015)	Cross sectional	USA	91.8	JIS	39.3 ± 10.4	131	1069	12	27.6 ± 4	123/79	109	90 ± 12	56 ± 14	90 ± 11	89
Demiralp *et al.*, (2021)	Cross sectional	Turkey	100%	NCEP/ ATPIII	-	19	43	44	28.2 ± 3.2	130 / 77	206 ± 106	102 ± 64	42 ± 8	99 ± 9	89
Donovan *et al.*, (2009)	Longitudinal	USA	100	NCEP/ ATPIII	39 ± 9	32	214	15	-	-	-	-	-	-	78
Hershey *et al.*, (2021)	Cross sectional	USA	95.2	JIS	47 ± 7.6	44	249	18	-	-	-	-	-	-	89
Leary *et al.*, (2020)	Cross sectional	USA	100	NCEP/ ATPIII	39.2 ± 9.3	20	47	43	30.6 ± 3	136/81	131	101 ± 27	47 ± 13	105 ± 12	67
Lee *et al.*, (2019)	Cross sectional	USA	97.4	NCEP/ ATPIII	-	38	273	14	-	-	-	-	-	-	89
Lee *et al.*, (2017)	Cross sectional	South Korea	100	NCEP/ ATPIII	42 ± 5	55	257	21	-	-	-	-	-	-	89
Leischik *et al.*, (2015)	Longitudinal	Germany	100	IDF	40.5 ± 9	12	97	12	25.9 ± 3.2	126/84	142 ± 75	66 ± 18	56 ± 13	-	78

Li *et al*., (2017)	Longitudinal	USA	93.1	NCEP/ ATPIII	46.9 ± 5.7	98	1099	9	-	-	-	-	-	-	89
Li *et al*., (2018)	Longitudinal	USA	92.2	NCEP/ ATPIII	37.2 ± 9.8	56	294	19	-	-	-	-	-	-	89
Kwon *et al*., (2019a)	Prospective	USA	100	NCEP/ ATPIII	39.5 ± 7.5	1173	6279	19	-	117/73	185 ± 136	92 ± 14	48 ± 12	-	78
Kwon *et al*., (2019b)	Prospective	USA	100	NCEP/ ATPIII	39.6 ± 7.5	1329	6947	19	-	117/73	184 ± 137	92 ± 14	48 ± 12	-	67
Moffatt *et al*., (2021)	Cross sectional	USA	98	NCEP/ ATPIII	42.4 ± 9.1	406	4513	9	29.1 ± 0.1	-	121 ± 2	96 ± 0.3	49 ± 0	-	100
Montazerifar *et al*., (2020)	Cross sectional	Iran	100	NCEP/ ATPIII	36.8 ± 7.5	47	140	34	27.4 ± 3.7	138/90	229 ± 91	91 ± 28	31 ± 4	96 ± 8	67
Seo *et al*., (2023)	Cross Sectional	USA	100	AHA/ NHLBI	-	66	114	58	-	-	-	-	-	-	89
Strauß *et al*., (2016)	Cross sectional	Germany	100	IDF	40.5 ± 9	14	97	14	25.9 ± 3.2	126/84	-	-	56 ± 13	90 ± 10	56
Supples *et al*., (2022)	Cross sectional	USA	95.1	NCEP/ ATP III	45	61	239	26	-	-	-	-	-	-	89
Title *et al*., (2008)	Longitudinal	Canada	100	NCEP/ ATPIII	50 ± 9	267	1417	19	-	-	-	-	-	-	100
Voss *et al*., (2017)	Cross sectional	USA	90	JIS	37.2 ± 9.5	112	600	19	27.4 ± 2	127/82	113	88 ± 8	46 ± 11	90 ± 10	78
Yang *et al*., (2014)	Cross sectional	USA	100	JIS	38 ± 9	227	780	29	-	-	-	-	-	-	78

AHA/NHLBI = American Heart Association and the National Heart, Lung, and Blood Institute; BMI = Body Mass Index; BP = Blood Pressure; FGB = Fasting Blood Glucose; HDL = High Density Lipoprotein Cholesterol; IDF = International Diabetes Federation; JIS = Joint Interim Statement; MetSyn = Metabolic syndrome; NCEP/ATP III = National Cholesterol Education, Adult Treatment Programme; RoB = Risk of Bias; TG = Triglycerides; WC = Waist Circumference
