# Supplementary figures and images for: The Prevalence of Metabolic Syndrome and Its Components in Firefighters: A Systematic Review and Meta-Analysis
Source: Int J Environ Res Public Health. 2023 Sep 23;20(19):6814. doi: 10.3390/ijerph20196814 (PMC10572458; doi:10.3390/ijerph20196814)

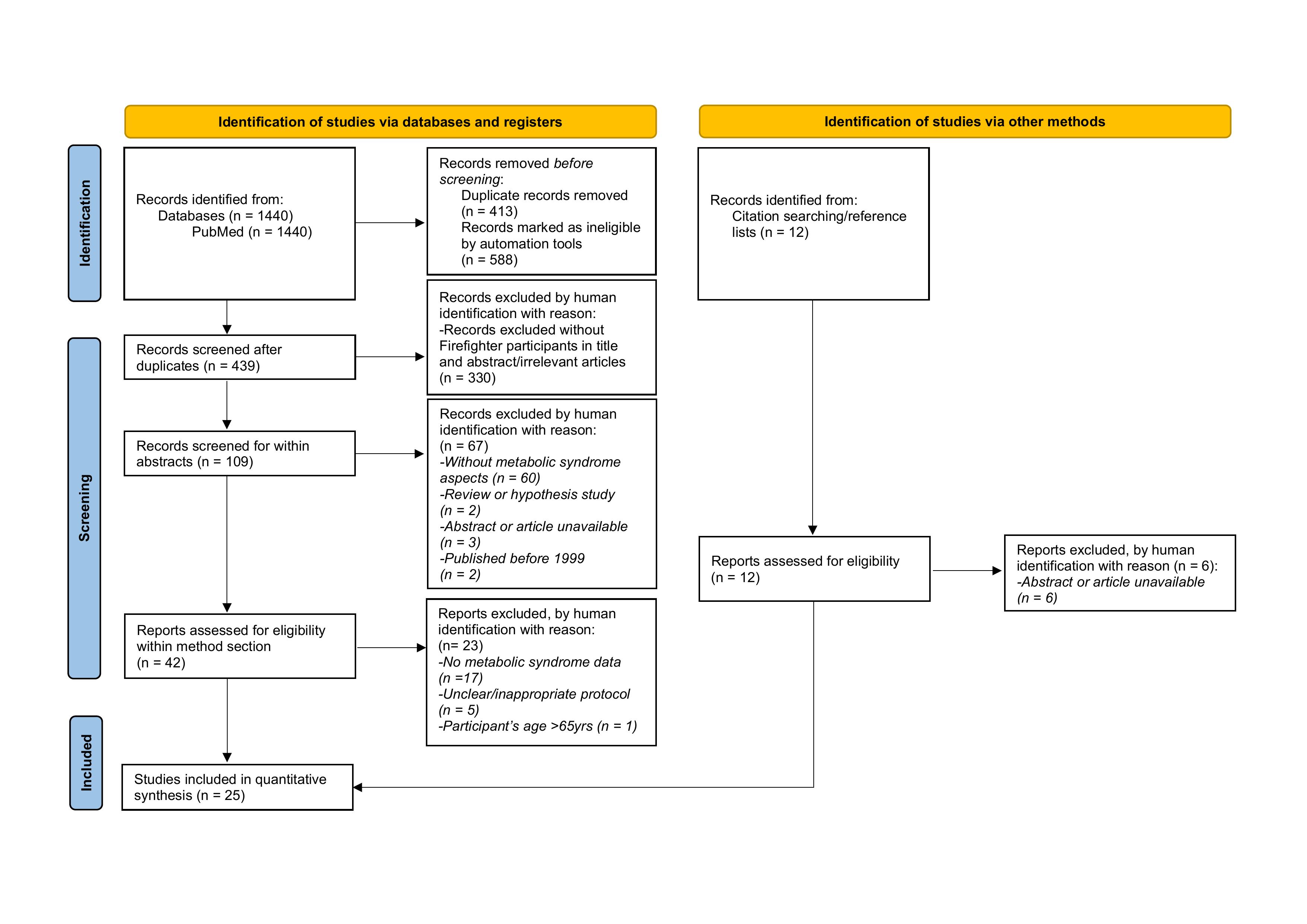

Supplement: Supplementary file 1 [file ijerph-20-06814-s001.zip › Supplementary Figure S1. PRISMA Diagram.jpg]

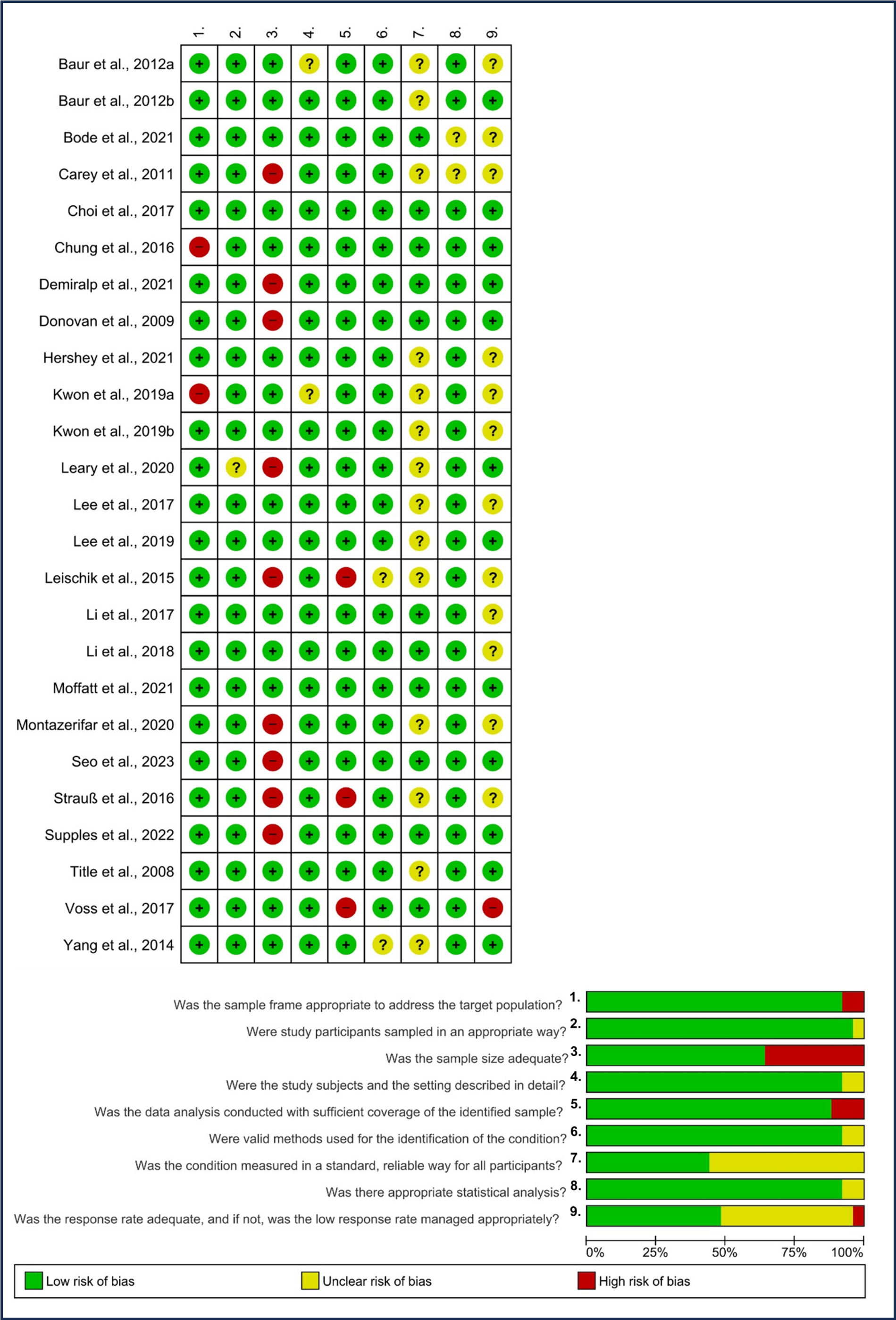

Supplement: Supplementary file 1 [file ijerph-20-06814-s001.zip › Supplementary Figure S2. Risk of Bias.png]

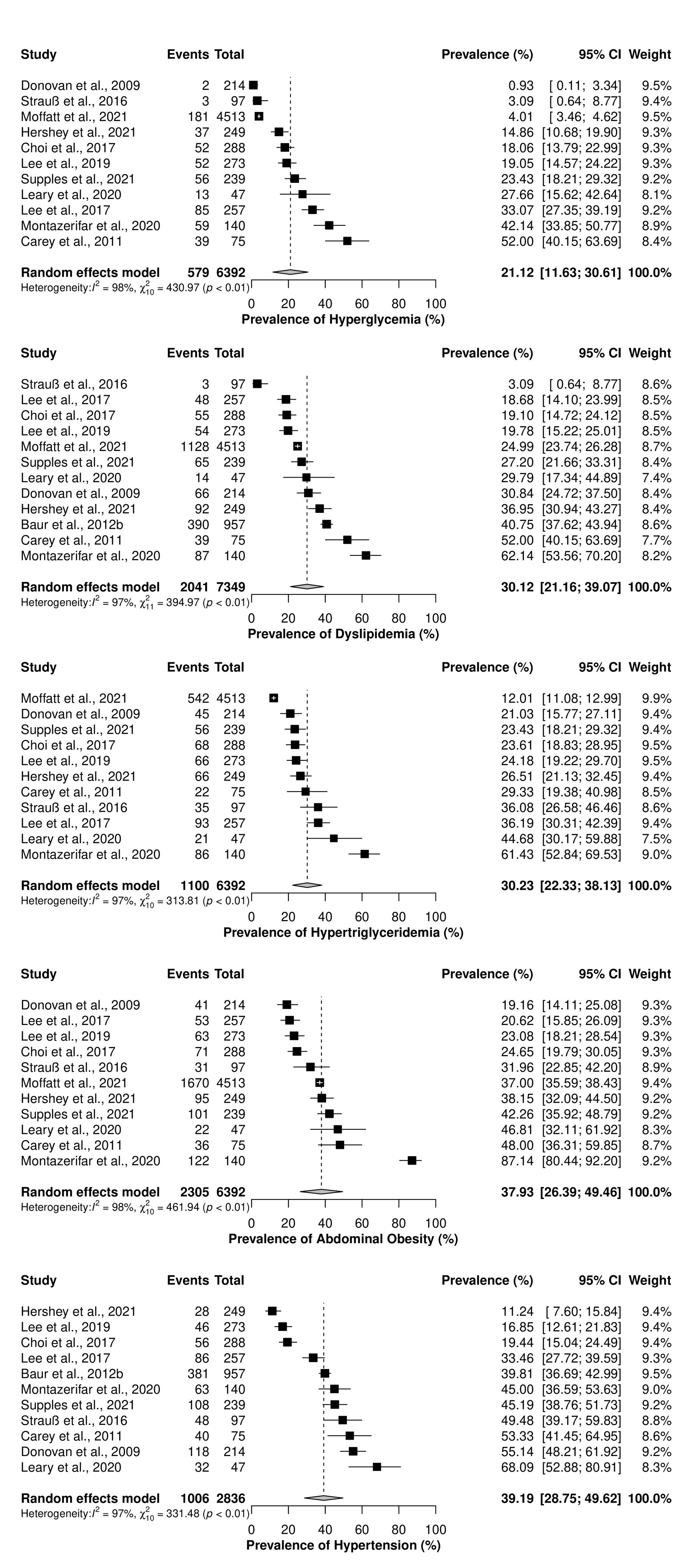

Supplement: Supplementary file 1 [file ijerph-20-06814-s001.zip › Supplementary Figure S3. Components of Metsyn Forest Plot.jpg]
